# Ayush Bala Rakshak Leham for Moderate Malnutrition in Children Aged 3 to 5 Years: Protocol for a Pilot Randomized Controlled Trial

**DOI:** 10.2196/93718

**Published:** 2026-07-06

**Authors:** Karthika Asokan Prabha, Kishor Gavali, Binitha P, Saniya CK, Suhas Chaudhary, Monika Kumari, Remya E, Prashant Shinde, Swati Sharma, Arunabh Tripathi, Sophia Jameela, Richa Singhal, Babita Yadav, Sarada Ota, Bhogavalli Chandra Sekhara Rao, Narayanam Srikanth, Rabinarayan Acharya

**Affiliations:** 1 National Ayurveda Research Institute for Panchakarma Central Council for Research in Ayurvedic Sciences Thrissur, Kerala India; 2 Maharao Shekhaji Regional Ayurveda Research Institute Central Council for Research in Ayurvedic Sciences Jaipur, Rajasthan India; 3 Regional Ayurveda Research Institute Central Council for Research in Ayurvedic Sciences Thiruvananthapuram, Kerala India; 4 Central Ayurveda Research Institute Central Council for Research in Ayurvedic Sciences Bhubaneswar, Odisha India; 5 Central Council for Research in Ayurvedic Sciences Janakpuri, New Delhi India; 6 Central Ayurveda Research Institute Central Council for Research in Ayurvedic Science Punjabi Bagh, New Delhi, Delhi India

**Keywords:** Ayurveda, Ayush-BR Leham, malnutrition, under-five children, World Health Organization Z score, WHO Z score, mortality

## Abstract

**Background:**

Malnutrition, a leading cause of mortality and morbidity in children aged <5 years, requires early detection and intervention. In Ayurveda, “Kumarasosha” closely corresponds to childhood malnutrition, with its pathophysiology and treatment principles offering a more precise and effective approach to management.

**Objective:**

This study is designed to evaluate the efficacy and safety of *Ayush Bala Rakshak (BR) Leham* in moderately malnourished children aged 3 to 5 years.

**Methods:**

This is a multicenter, open-label, randomized controlled, 2-group pilot study designed to enroll 200 moderately malnourished children, 100 from each participating study center of the Central Council for Research in Ayurvedic Sciences. The children will be screened for moderate malnutrition using the World Health Organization weight-for-age *Z* score through the AnthroCal app developed by the All India Institute of Medical Sciences, New Delhi. After obtaining consent from parents, the participants will be randomized and allocated to 2 groups. The study group will receive *Aravindasava*, 5 mL twice daily after food for 4 weeks, followed by *Ayush BR Leham*, a nutritional supplement, 5 g twice daily before food from weeks 5 to 12. The control group will receive *Aravindasava* for 4 weeks and thereafter receive no intervention for 8 weeks. Both groups will be advised to follow a home-prepared diet during the study period. A follow-up assessment will be conducted after 4 weeks. The primary outcome is a change of ≥0.5 in the World Health Organization weight-for-age *Z* scores, and the secondary outcomes are changes in anthropometric measurements, the frequency of illnesses assessed using a predesigned table, and safety parameters assessed using biochemical measures.

**Results:**

The study received funding in March 2023. Participant enrolment commenced on December 16, 2023 and has been completed. After data analysis, the study findings are expected to be submitted for publication in December 2026.

**Conclusions:**

The study will investigate the efficacy and safety of *Ayush BR Leham* in moderately malnourished children aged 3 to 5 years. If the intervention is found to be effective, the findings may help incorporate Ayurvedic guidelines into standard treatment protocols for children with moderate malnutrition.

**Trial Registration:**

Clinical Trials Registry of India CTRI/2023/07/055188; https://tinyurl.com/3arpbecm

**International Registered Report Identifier (IRRID):**

DERR1-10.2196/93718

## Introduction

### Background and Rationale

Malnutrition remains one of the leading public health challenges worldwide, particularly affecting children aged <5 years. In India, the prevalence of malnutrition continues to be high despite significant economic growth and the implementation of various national nutritional programs. According to the National Family Health Survey (NFHS-4, 2015-2016), 38.4% of children aged <5 years were stunted, 35.7% were underweight, and 21% were wasted [1]. Regional data further indicate that the prevalence of underweight status ranges from 39% to 75% [2]. Malnutrition in children arises from multiple factors, such as inadequate dietary intake, frequent infections, poor sanitation, lack of access to quality health care, and suboptimal infant and young child feeding practices. It impairs immune function, increases vulnerability to infections, and results in long-term deficits in physical and cognitive development [3]. Malnutrition is also a significant contributor to mortality among children aged <5 years, accounting for more than one-third of deaths in this age group [4]. Early identification and timely intervention are crucial for improving outcomes in affected children.

Ayurveda offers a holistic and well-structured understanding of pediatric nutritional disorders. Conditions such as *Kumarasosha*, *Phakka*, *Parigarbhika*, and *Karshya* describe states of compromised nutrition in children, in which the key pathophysiological mechanisms, such as *Agnimandya* (impaired digestion and metabolism) and *Srotorodha* (obstruction in body channels), lead to *Dhatu Sosha* (undernourishment of body tissues) [5-8]. The Ayurvedic approach to managing such conditions emphasizes the correction of *Agni* and the clearing of *Srotas* (body channels) using *Deepana* and *Pachana* (digestive stimulants and carminatives). This is followed by *Brimhana* therapy (nourishment) to promote healthy tissue development [9]. Restoration of *Dhatu Parinama* (tissue transformation) ultimately enhances *Ojas*—the essence of vitality and immunity—thereby ensuring better resistance to disease and supporting optimal growth and development in children [10]. Ayurveda has a diverse pharmacopoeia of single and compound formulations tailored to specific age groups. Pediatric formulations are designed not only for efficacy but also for palatability and compliance. Dosage forms such as *Avaleha* (herbal jams) and *Asava* (fermented herbal decoctions) are commonly used in children. *Aravindasava*, an *Asava* preparation with *Deepana* and *Pachana* properties, is frequently prescribed in pediatric care as a corrective therapy for *Agnimandya*.

Although various national-level interventions are in place, undernutrition in Indian children remains a significant public health burden [2]. Ayurveda, with its individualized and holistic approach, offers promising alternatives or complementary strategies for managing pediatric malnutrition. *Ayush Bala Rakshak (BR) Leham*, formulated based on classical Ayurvedic principles and contemporary clinical needs, has the potential to improve nutritional outcomes in moderately malnourished children. This multicenter, randomized controlled pilot study aims to evaluate the efficacy and safety of *Ayush-BR Leham* in children aged 3 to 5 years with moderate malnutrition. The findings will contribute to the evidence-based integration of Ayurveda into pediatric nutritional management protocols.

*Ayush-BR Leham* is a nutritional supplement that contains herbs with *Deepana*, *Pachana*, *Srotoshodhana* (channel-cleansing), *Brimhana*, *Anulomana* (regulating *Vata*), and *Rasayana* (rejuvenating) properties. These pharmacological actions are aimed at enhancing digestion, nutrient assimilation, and tissue nourishment, thereby contributing to improved immunity and weight gain in malnourished children. This nutritional supplement combines both light and heavy components to strike a balance between digestibility and nourishment. It is prepared by triturating powdered herbs in honey, avoiding *Agnisamparka* (direct heat) to preserve the bioactivity of the ingredients. In this study, *Aravindasava* is administered to both the intervention and control groups as a standard care measure to prime the digestive system. *Ayush-BR Leham*, administered additionally to the intervention group, is expected to enhance therapeutic outcomes by synergistically supporting digestion, absorption, immunity, and growth. Both groups are advised to maintain a nutritious home-prepared diet during the study period.

### Objective

The study’s objective is to evaluate the therapeutic efficacy and safety profile of *Ayush-BR Leham* in children aged 3 to 5 years with moderate malnutrition.

## Methods

### Trial Design

**Figure 1:**
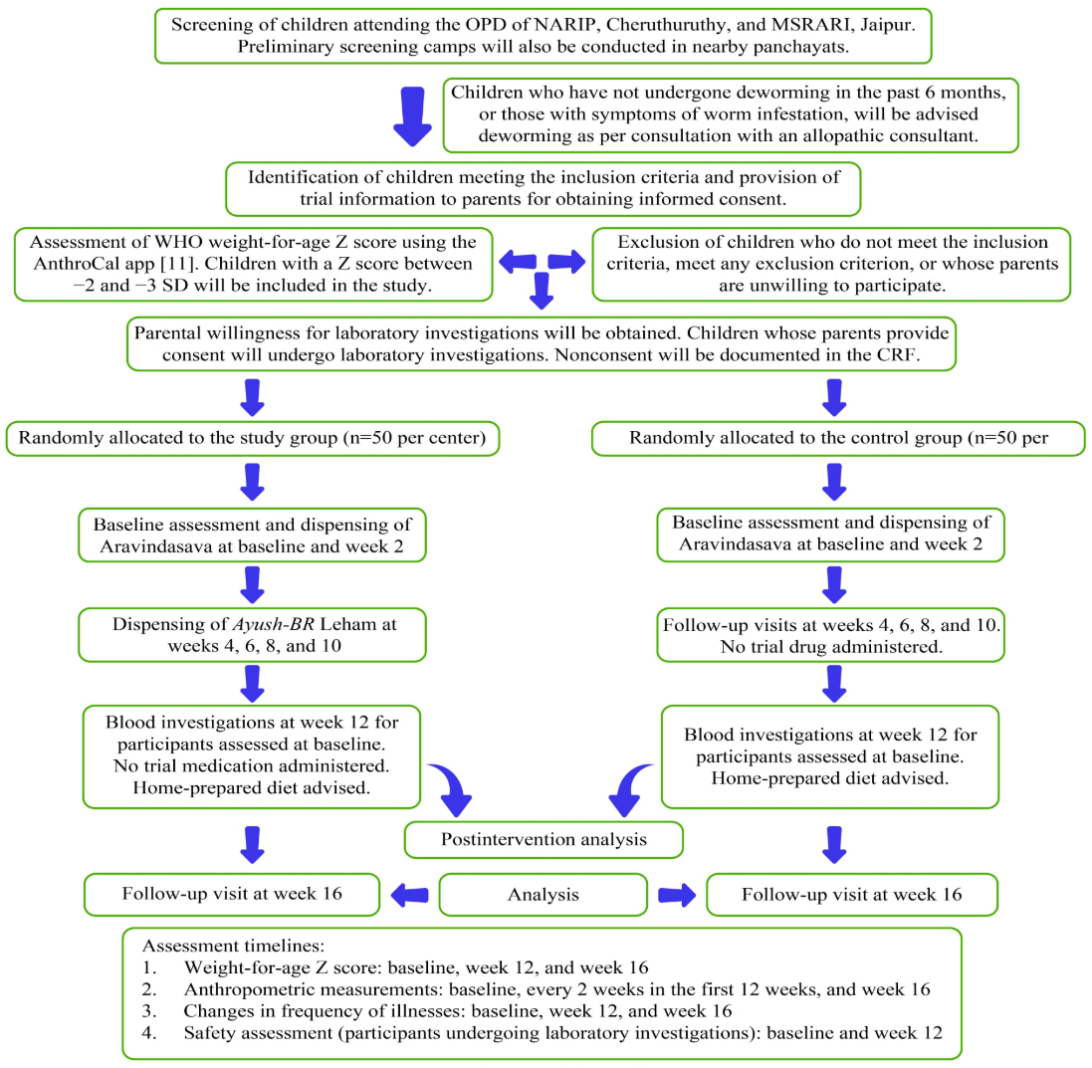
Flowchart of the study plan. BR: Bala Rakshak; CRF: case report form; MSRARI: Maharao Shekhaji Regional Ayurveda Research Institute; NARIP: National Ayurveda Research Institute for Panchakarma; OPD: outpatient department; WHO: World Health Organization.

The study is planned as a multicenter, open, randomized controlled, 2-group pilot study designed to investigate the therapeutic efficacy and safety profile of *Ayush-BR Leham* in managing moderate malnutrition in children aged 3 to 5 years. After obtaining written informed consent from the parents, the participants fulfilling the inclusion criteria will be randomized and assigned to the study and control groups in a 1:1 ratio. Both the study and control groups will be administered *Aravindasava* for 4 weeks to provide an initial correction of digestive function. Then, for a period of 8 weeks, *Ayush-BR Leham* will be administered to the study group, and the control group will be followed without any intervention. Both groups will undergo a follow-up period of 4 weeks. Throughout the study, both groups will be advised to strictly follow a home-prepared diet. The study protocol is reported in accordance with the SPIRIT (Standard Protocol Items: Recommendations for Interventional Trials) guidelines. The flowchart of the study plan is shown in Figure 1 [11].

### Study Setting

The research study will be conducted at the outpatient department of the National Ayurveda Research Institute for Panchakarma, Cheruthuruthy, Kerala, and the Maharao Shekhaji Regional Ayurveda Research Institute, Jaipur, Rajasthan.

### Eligibility Criteria

Children aged 3 to 5 years with a weight-for-age measurement between −2 and −3 SD according to the World Health Organization (WHO) weight-for-age growth standards are included in the study. Children should be free from edema, and their parents or caregivers should be willing to provide written informed consent for their children’s participation. Children with diagnosed serious health conditions such as heart disease, lung disease, concurrent hepatic disorder, renal disorder, congenital anomalies, inborn errors of metabolism, malignancy, tuberculosis, or HIV, as well as children with acute infections, including respiratory tract infections, diarrhea, or fever, will be excluded from the study. Children with known allergies to the trial medication or its components, children who have participated in other clinical trials within the past 6 months, and children with any other condition deemed by the investigator to pose a risk to study validity will also be excluded.

### Interventions

The drug *Aravindasava* has been procured from Indian Medicines Pharmaceutical Corporation Limited, a Good Manufacturing Practice–certified pharmaceutical company in Almora, Uttarakhand, with a quality assurance certificate ensuring compliance with the Ayurveda Pharmacopoeia of India standards [12]. The trial drug *Ayush-BR Leham* was prepared at the pharmacy of the Central Ayurveda Research Institute, Kolkata, as per the standard operating procedures developed by the Central Council for Research in Ayurvedic Sciences (CCRAS). The study drugs will be stored in a dark, cool, and dry place. The investigators will administer *Aravindasava* in both groups for 4 weeks at a dose of 5 mL twice daily after food, followed by *Ayush-BR Avaleha* in the study group at a dose of 5 g twice daily before food for 8 weeks. There will be no intervention in the control group after *Aravindasava* administration.

Both groups receive *Aravindasava*, ensuring that no group is disadvantaged in the early, critical phase, during which correction of digestion, absorption, and assimilation is necessary. All participants will receive standard nutritional care and public health interventions available to the general pediatric population with malnutrition during the study period. These include regular nutritional counseling by Accredited Social Health Activist workers or *Anganwadi* workers through government initiatives, age-appropriate dietary guidance from the study pediatrician, health check-ups and monitoring by the research team, management of infectious diseases that develop during the study, immunization, and routine health care. Both groups are advised to follow a home-prepared diet throughout the study. A home-prepared diet refers to age-appropriate, nutrient-dense meals that the family provides at home following personalized dietary counseling by the study team. It represents optimized nutrition within the family’s resources and cultural context. Compliance with the home-prepared diet will be recorded in the case report form (CRF).

### Outcome Measures

The primary outcome measure is the proportion of participants attaining a change in the weight-for-age *Z* score (WAZ) by ≥0.5, as per WHO reference standards, from baseline to weeks 12 and 16. The secondary outcome measures include changes in anthropometric measurements (weight in kg, height in cm, and midupper arm circumference in cm), which will be assessed every 2 weeks for 12 weeks, followed by assessment at week 16. Change in the frequency of illnesses will be assessed at baseline, week 12, and week 16 using a predesigned table that records the frequency and duration of illnesses. Assessment of safety (complete blood count [CBC], liver function test [LFT], renal function test [RFT], and serum electrolytes) will also be performed at baseline and week 12 in a small proportion of participants whose parents provide consent.

### Safety Monitoring

The safety population is defined as all randomized participants who received at least 1 dose of the study intervention or were allocated to the control group and underwent at least 1 postbaseline visit. This population will be used for all safety analyses, regardless of protocol adherence, study completion status, or parental consent to laboratory investigations. Adverse events will be actively monitored at every study visit through symptom inquiry from parents or guardians, physical examination (temperature, pulse, respiratory rate, abdominal examination, and anthropometric measurements), review of concomitant medications, and laboratory assessments in a small group of participants. Laboratory investigations will also be conducted when there is a clear clinical need, rather than routinely for all participants. Specifically, if a child shows signs of liver disease, such as jaundice, LFTs will be performed within 48 to 72 hours after discussing the need for testing with parents. Similarly, for suspected kidney problems, such as reduced urine output, hematuria, or swelling, RFTs will be performed within the same time frame. If there are suspected blood-related issues, such as pallor or bleeding, a CBC and coagulation profile will be performed within 24 to 48 hours. For suspected metabolic or endocrine effects, tests such as fasting glucose, electrolytes, or thyroid function tests may be performed within 72 hours, depending on the symptoms. For a small group of willing participants, baseline and week 12 laboratory tests, including CBC, erythrocyte sedimentation rate, LFT, RFT, and serum electrolytes, will be performed, with separate consent obtained from parents. In all cases, parents will be fully informed about the necessity of the tests, and their concerns will be addressed to ensure they are comfortable with the process.

### Sample Size

The sample size for the study has been calculated assuming that 60% of participants in the intervention arm and 30% of participants in the control arm will achieve a change in *Z* score of ≥0.5 according to the WHO weight-for-age growth standards. With a 95% CI and 80% power, 42 participants per group need to be enrolled in the trial. After accounting for an attrition rate of 20%, the sample size for the study has been finalized as 50 per group. Therefore, a total of 100 participants per center will be enrolled in the trial.

### Recruitment

Awareness sessions and one-on-one interactions will be held for outpatients to facilitate recruitment. Screening camps will also be conducted in local panchayats to boost enrollment and support the timely completion of recruitment.

### Randomization and Allocation

After getting consent from parents or guardians, participants will be randomized and allocated to the study group or control group in a ratio of 1:1 with the help of serially numbered opaque sealed envelopes. Due to the nature of the trial, participant and investigator blinding is not feasible.

### Concomitant Medication

Parents will be advised against self-medicating their children and will instead be encouraged to consult the investigator for any symptoms. The investigating physician will document any prescribed medications.

### Ancillary and Posttrial Care

All clinical trial participants will receive appropriate ancillary care, including management of any adverse events or unrelated health issues that arise during the study. Posttrial participants will be given appropriate treatment for management of their condition, if required, or will be referred to appropriate health care services, in accordance with ethical standards.

### Data Collection

#### Baseline Assessments

At baseline, after recording the medical history, other details, such as demographic characteristics, Ayurveda parameters, anthropometric measurements, the WAZs, and illnesses during the past 3 months, will be collected. Laboratory investigations will be performed for participants whose parents provide consent.

#### Follow-Up Assessments

All participants will undergo follow-up assessments on days 14, 28, 42, 56, 70, 84, and 112. Concomitant medication, rescue medication, drug compliance, and adverse drug reactions or adverse drug events will be assessed during the follow-up visits. Laboratory investigations will be performed at the day 84 visit for those who underwent baseline laboratory assessments (Table 1).

**Table 1:** Study schedule.

Assessments	Screening or baseline	Follow-up		
		Weeks 2-10 (days 14, 28, 42, 56, and 70)	Week 12 (day 84)	Week 16 (day 112)
Informed consent from parent	✓			
Laboratory investigations (for those who are willing)	✓		✓	
Demographic characteristics and medical history	✓			
Weight-for-age *Z* score	✓		✓	✓
Anthropometric measurements (weight, height, midupper arm circumference, and BMI)	✓	✓	✓	✓
Assessment of illness frequency	✓		✓	✓
*Dosha*, *dhatu*, and *srotopareeksha* assessment	✓			
Assessment of concomitant medications		✓	✓	✓
Assessment of rescue medications		✓	✓	✓
Assessment of adverse drug reactions and adverse events		✓	✓	✓
Drug compliance assessment		✓	✓	
Dispensing of trial drug and compliance reporting form	✓	✓		

### Trial Status and Dissemination Policy

Recruitment of study participants began in December 2023, shortly after procurement of the trial drug. The research findings will be disseminated through top-tier national and international peer-reviewed journals.

### Participant Withdrawal Guidelines

Participants will be withdrawn from the trial if they experience a serious adverse event, regardless of whether it is related to the trial drug or if they experience an adverse drug reaction that requires discontinuation of the trial medication. Acute illness requiring inpatient treatment during the study period, worsening of the existing condition, voluntary withdrawal from the study by parents, and drug compliance of less than 80% will also result in withdrawal from the study. The reason for withdrawal will be documented in the CRF, dated, and signed. Participants withdrawn from the trial will undergo a final examination if possible. If participants leave against medical advice, a final telephone interview will be conducted with the parents to assess the participant’s health status from the point of view of drug safety and the validity of study results.

### Data Management

Data will be collected using paper and electronic CRFs, managed by the principal investigator, and stored securely. Participants will be assigned a unique enrollment number. Data entry will prioritize accuracy, and the data will be stored for at least 5 years after study completion.

### Protocol Amendments

The trial is being conducted according to the approved protocol. Any deviation required in the protocol, including changes in the interventions or methods, will be reported to the sponsors and the institutional ethics committee (IEC), along with justification. Protocol amendments will be implemented only after IEC approval, following standard procedures.

### Statistical Methods

The primary outcome of the study, namely, the proportion of participants achieving an improvement in the WAZ of ≥0.5, will be analyzed using an intention-to-treat approach. Between-group comparisons for the primary binary outcome will be performed initially using the chi-square test, followed by multivariate logistic regression to estimate adjusted effect sizes after controlling for important covariates, including study center, baseline, WAZ, age, sex, and other clinically relevant variables. This will be done in the following steps:

- Step 1: an unadjusted between-group comparison will be performed using the chi-square test to examine the crude difference in the proportion of participants achieving the primary outcome between the intervention and control groups. This provides transparency and allows readers to evaluate the raw effect.
- Step 2: multivariate logistic regression will be used to estimate the adjusted odds ratio and 95% CI, while controlling for the following prespecified covariates: study center, baseline WAZ (continuous), age (months), sex, baseline nutritional status category, socioeconomic status, and educational status of parents. The adjusted analysis represents the primary analytic result and will be emphasized in reporting and interpretation.

For the secondary outcomes, within-group pre-post changes will be examined using a paired *t* test or Wilcoxon signed-rank test, as appropriate, and will be reported for descriptive purposes. For parameters assessed at multiple follow-up time points, repeated-measures ANOVA or the Friedman test will be applied to assess temporal trends. All analyses will be performed using SPSS (version 29.0; IBM Corp), and statistical significance will be assessed at α=.05 (2-tailed).

Participants who withdraw, are lost to follow-up, or do not complete the week 12 assessment will be documented, and reasons for dropout will be recorded. Dropout rates and reasons will be presented by study group in the CONSORT (Consolidated Standards of Reporting Trials) flowchart.

### Data Monitoring

CCRAS, the trial’s sponsor and coordinating center, will conduct periodic site monitoring through a committee of experts and a biostatistician to ensure compliance with Good Clinical Practice and the study protocol.

### Ethical Considerations

The IEC at National Ayurveda Research Institute for Panchakarma, Cheruthuruthy (F.No. 8/16/2023/NARIP/Tech meeting/2506; March 31, 2023), and the IEC at Maharao Shekhaji Regional Ayurveda Research Institute, Jaipur (F.5/Lab/Project/Ethics/2007-08CRAI-JPR/Part-1/455; May 3, 2023), approved this study. The trial was prospectively registered with the Clinical Trials Registry of India (CTRI/2023/07/055188) on July 13, 2023. This trial is conducted in accordance with the Declaration of Helsinki, the Indian Council of Medical Research guidelines 2017, and CCRAS Research Policy. Informed consent will be obtained from parents by the investigators before trial participation, ensuring that the parents’ right to autonomy is upheld. Participant confidentiality will be maintained throughout all phases of the study, with access to data granted only to study personnel, sponsors, and the monitoring team. The IEC has approved the study protocol and the written informed consent form, ensuring the confidentiality and privacy of participants’ personal and medical information.

## Results

The study received funding in March 2023. Participant enrolment commenced on December 16, 2023 and has been completed. The study is ongoing at 2 CCRAS centers. After data analysis, the study findings are expected to be submitted for publication in December 2026.

## Discussion

### Anticipated Findings

Malnutrition stunts the growth and potential of children, ultimately hindering a nation’s economic progress and prosperity. Reinforcing public health interventions in children aged <5 years, facilitating early diagnosis, ensuring effective implementation, and evaluating strategies and measures aimed at socioeconomic development are imperative for the successful management of children aged <5 years’ malnutrition in India [2]. A recent study revealed that mild underweight variability has a stronger association with child mortality rates than severe underweight variability [13]. Hence, mild to moderate malnutrition should not be overlooked, and its management should be prioritized to prevent long-term consequences.

Most conventional strategies emphasize nutritional rehabilitation primarily through supplementation. However, they may not adequately address the underlying digestive and metabolic disturbances that can limit nutrient use [14]. Emerging evidence from gut microbiome research shows that malnourished children often have immature or dysbiotic gut microbiota [15]. Additionally, dietary patterns in children significantly influence gut microbiome composition, thereby affecting nutrient metabolism and energy harvest [16,17]. Conventional supplements do not help restore a healthy gut microbiota, potentially limiting long-term recovery from malnutrition. This protocol addresses this gap by incorporating an initial phase of digestive correction before the initiation of nourishing therapy. According to Ayurveda, every disease originates from weak digestive power. Malnutrition in children is correlated with *Kumarasosha* a condition resulting from various faulty dietary etiologies affecting digestion. These etiologies include undernutrition, the consumption of junk foods, and incompatible diets. Such practices, if continued, primarily weaken digestion and lead to the formation of toxic metabolites (*ama*), which accumulate in the body channels, obstruct them, and affect tissue transformation. At this juncture, if nourishing drugs or dietary interventions are administered, they can lead to improper digestion, which may further weaken digestion and increase the accumulation of *ama*, thereby obstructing the channels and perpetuating this vicious cycle. Ayurveda emphasizes the primary correction of digestive fire (*agni*) and the clearance of toxic metabolites and channel obstructions before starting nourishing therapy. This first line of management is essential for creating a suitable platform for nourishing therapy. The second line of management is nourishing therapy itself.

The *Kashyapa Samhita*, a classical textbook of Ayurvedic pediatrics, defines “Lehana” as a formulation that is heavy to digest but nourishing and is recommended for emaciated children [18]. *Ayush-BR Leham*, the trial drug, is one such nutritional supplement. The nutrition supplied after digestion correction is expected to be properly digested and assimilated by the body, supporting healthy weight gain [19]. Studies support that intestinal dysfunction, inflammation, and environmental enteric dysfunction can impair nutrient absorption despite adequate intake from fortified foods [20]. Hence, the sequential treatment approach is conceptually robust, as it aims to optimize digestive and metabolic functions before nutritional supplementation, thereby enhancing nutrient bioavailability and assimilation [21]. Such a strategy is likely to promote more effective and sustained recovery, rather than transient weight gain alone. This study design thus offers more treatment responsiveness and reduces the likelihood of incomplete recovery observed in standard supplementation-based interventions.

This study is designed as an exploratory randomized controlled trial to evaluate the efficacy of the nutritional supplement, *Ayush-BR Leham*, in moderately malnourished children. It is hypothesized that the trial intervention will result in a clinically meaningful improvement in nutritional status, reflected by an increase of ≥0.5 in WAZ among moderately malnourished children. Additionally, the intervention is expected to improve anthropometric parameters and reduce illness frequency compared with the control group.

The study has several strengths, including a randomized controlled design, the use of standardized and clinically relevant outcome measures such as WAZ, and repeated follow-up assessments that enable evaluation of trends over time. WAZ is a well-established indicator of child mortality and is sensitive to changes associated with nutritional interventions. Previous trials have demonstrated that improvements in WAZ reflect meaningful recovery and improved clinical outcomes [22,23]. Even mild improvements in WAZ following a short-term intervention with a nutritional supplement signify a positive shift in overall nutritional status and reduced morbidity. Additionally, a *Z* score ≥0.5 exceeds the normal day-to-day variation expected due to fluctuations in hydration status, illness, or measurement errors [24].

The intervention is based on a rational approach that addresses both nutritional deficiency and the underlying pathology, adding novelty to the study. However, the design does not permit complete isolation of the independent effects of the intervention components due to the initial common treatment phase. Although both groups are advised to follow a home-prepared diet, variations in dietary composition, quantity, and adherence at the household level may introduce residual confounding. However, the randomized design is expected to distribute such dietary variations equally between groups, thereby minimizing their impact on the comparative outcome. Despite these limitations, the study is expected to generate preliminary evidence to inform larger confirmatory studies across diverse geographic and socioeconomic settings, thereby enhancing external validity. Implementation research exploring its feasibility within community-based platforms, such as Integrated Child Development Services and *Poshan Abhiyan*, could support policy-level adoption if efficacy is established.

### Future Prospects

*Ayush-BR Leham* is expected to improve the WAZ (WHO), anthropometric measurements, and the frequency of illness. The trial is anticipated to yield crucial data on the efficacy and safety of this nutritional supplement. The results of this study may give scientific insights for the development of strategies to manage moderate malnutrition in children. If the intervention is found to be effective, the findings may help in the incorporation of Ayurvedic guidelines into standard treatment protocols for children with moderate malnutrition.

## Appendix

Multimedia Appendix 1 Peer-reviewer report from the Project Evaluation Monitoring Committee, Central Council for Research in Ayurvedic Sciences, Ministry of Ayush, Government of India.

## Abbreviations

BR: Bala Rakshak
CBC: complete blood count
CCRAS: Central Council for Research in Ayurvedic Sciences
CONSORT: Consolidated Standards of Reporting Trials
CRF: case report form
IEC: institutional ethics committee
LFT: liver function test
RFT: renal function test
SPIRIT: Standard Protocol Items: Recommendations for Interventional Trials
WAZ: weight-for-age Z score
WHO: World Health Organization

## Data Availability

As this is an ongoing study, the findings will be disseminated through publication in peer-reviewed scientific journals and presentation at national and international conferences, seminars, and academic forums after study completion. The data generated during the study will not be publicly available at this stage owing to the ongoing nature of the research and applicable institutional and governmental regulations. However, deidentified participant data underlying the study findings may be made available upon reasonable request to the corresponding author after publication of the study outcomes, subject to review and approval by the competent authority and in accordance with applicable Government of India norms, institutional policies, ethical guidelines, and data-sharing regulations. Any shared data will be provided only after ensuring confidentiality and protection of participant privacy.
