# Supplementary material for: Ayush Bala Rakshak Leham for Moderate Malnutrition in Children Aged 3 to 5 Years: Protocol for a Pilot Randomized Controlled Trial
Source: JMIR Res Protoc. 2026 Jul 6;15:e93718. doi: 10.2196/93718 (PMC13386115; doi:10.2196/93718)
Supplement: Multimedia Appendix 1 [file resprot_v15i1e93718_app1.pdf]

केन्द्रीय आयुर्वेदीय विज्ञान अनुसंधान परिषद्
(भारत सरकार स्वास्थ्य एवं परिवार कल्याण मन्त्रालय के अधीन गठित स्वशासी निकाय)
जवाहर लाल नेहरू भारतीय चिकित्सा एवं होम्योपैथी अनुसंधान भवन
नं. ६१-६५, इन्स्टिट्यूशनल एरिया, सम्मुख 'डी' ब्लाक, जनकपुरी, नई दिल्ली-११००५८
CENTRAL COUNCIL FOR RESEARCH IN AYURVEDIC SCIENCES
(An autonomous organisation under Ministry of Health & Family Welfare, Govt. of India)
Jawahar Lal Nehru Bhartiya Chikitsa Evam Homoeopathy Anusandhan Bhawan
No.61-65, Institutional Area, Opp.'D' Block, Janakpuri, New Delhi-110058

Gram : **"AYUSH"**
Fax : 28520748
EPBX
28525852, 28520501
28522524, 28525831
28525862, 28525883
28525897

F.No.-3-34/2021-CCRAS/Admn. /3370 **Date:** 20 SEP 2022

Subject: Minutes of 2nd Meeting of Project Evaluation Monitoring Committee (PEMC) held on 13th & 14th September, 2022 in the Committee Room, CCRAS Hqrs., New Delhi.

Sir,

Please find enclosed herewith the Minutes of 2nd Meeting of Project Evaluation Monitoring Committee (PEMC) [after revised CCRAS Research Policy] held on 13th & 14th September, 2022 in Hybrid Mode in the Committee Room, CCRAS Hqrs., New Delhi duly approved by the Committee for kind information and record.

Yours faithfully,

(Dr. N. Srikanth)
Deputy Director General & Member Secretary

To,

1.	**Prof. (Dr.) Rabinarayan Acharya, Director General,** CCRAS, New Delhi	**Chairman**
2.	**Dr. Atul Juneja,** Former Scientist E, ICMR, New Delhi	**Member**
3.	**Mr. Raj Kumar,** Director (IFD), Ministry of Ayush, New Delhi.	**Member**
4.	**Dr. Sunita Garg,** Former Principal Scientist, NISCAIR , CSIR, New Delhi	**Member**
5.	**Prof. (Dr.) Suresh Kumar,** Dept. of Botany, Ramjas College, Delhi	**Member**
6.	**Dr. Rajeev Sharma,** Former Director, PCIM, Gaziabad, U.P	**Member**
7.	**Dr. Sharad Srivastav,** Senior Principal Scientist and Head,Pharmacognosy Division, NBRI, Lucknow	**Member**
8.	**Prof. Yogender Pal Khasa,** Department of Microbiology, University of Delhi, Delhi	**Member**
9.	**Dr Sonia Khatter,** Professor(Microbiology), ESIC, Delhi	**Member**
10.	**Dr. Gian Singh,** Former Scientist 'F' and Head, NISCAIR, Delhi	**Member**
11.	**Dr. Virendra Singh Rana,** Principal Scientist, IARI , New Delhi	**Member**
12.	**Dr. S. K. Maulik,** Former professor, AIIMS, Delhi	**Member**
13.	**Dr. Sharad Wakode,** Prof. (Pharmaceutical Chemistry), DIPSAR, Delhi	**Member**
14.	**Dr.Galib,** Associate Professor, AIIA, New Delhi	**Member**
15.	**Prof. B.J.Patgiri,** (Dean Research), ITRA, Jamnagar, Gujarat	**Member**
16.	**Prof. Anil Singh,** Department of Dravyaguna, IMS, BHU, Varanasi	**Member**
17.	**Dr.Sudipt Rath,** Associate Professor, Dept. of Dravyguna, NIA, Jaipur	**Member**
18.	**Dr.M.M.Padhi,** Ex-DDG, CCRAS, Bhubaneswar	**Member**
19.	**Dr. Asit K. Panja,** Associate Professor, NIA, Jaipur	**Member**
20.	**Dr. Arun Gupta,** Professor Panchakarma, CBPACS, Najafgarh, Delhi	**Member**
21.	**Prof. A.C.Kar,** Vikriti Vigyan, IMS, BHU,Varanasi	**Member**

Copy to:

- All Programme Officers and Nodal Officers with request to process the files/e-files for necessary sanction ensuring the necessary compliance.
- The dealing officer from Technical and Admn. Section with direction to process the file for each project separately through file concerned/e-office.
- Directors/ In-charges of CCRAS peripheral Institutes/Centres/Units for taking necessary action.
- Ad.O (Projects) for necessary action related to sanction of the approved projects
- Accounts Officer, CCRAS and Budget section for record.
- P.S. to Director General/P.S. to DDG

Deputy Director General & Member Secretary

CENTRAL COUNCIL FOR RESEARCH IN AYURVEDIC SCIENCES

Minutes of 2nd Meeting of Project Evaluation Monitoring Committee (PEMC) held on 13th & 14th September, 2022 in the Committee Room, CCRAS Hqrs., New Delhi

The 2nd Meeting of Project Evaluation Monitoring Committee (PEMC) [after Revised CCRAS Research Policy] held on 13th & 14th September, 2022 in Hybrid mode under the Chairman ship of DG-CCRAS in the Committee Room CCRAS Hqrs, New Delhi. The Details of the members and Council officers attended is appended at **Annexure -I**.

At the outset, Prof. (Vaidya) Rabinarayan Acharya, Director General, CCRAS and Chairman of PEMC, welcomed the members and invited experts. After that, Dr. N. Srikanth, Dy. Director General, CCRAS and Member Secretary of PEMC briefed about the purpose of the meeting and briefed about the Agenda items proposed for this discussion.

Dr. N. Srikanth, Dy. Director General, CCRAS and Member Secretary of PEMC proposed discussions on Agenda of the meeting. First of all, he briefed about the progress of all the completed/ongoing projects for appraisal of the Committee under Agenda- 1. After that, he informed that the new proposals viz. 4 projects related to Literary Research, 18 projects related to Medicinal Plant Research, 13 projects related to Drug Standardization, 5 projects related to Pharmaceutical Research, 4 projects related to Pharmacology Research and 20 projects related to Clinical Research are proposed to put before the Committee. The details are as under:

Agenda 1: Appraisal of completed/ongoing projects: Total 233 completed research projects during last five years including current year and 164 ongoing research projects have been discussed and appraised by the committee. The details are annexed at **Annexure – II.**

Agenda 2: Literary Research: The proposals of **4** research projects were placed before the committee. After detailed discussion, **1** proposal with **Agenda items no. 2.1** were approved without any modification and **3** projects with **Agenda items no. 2.2, 2.3 & 2.4** were approved by the committee with the certain modifications and suggestions. The detailed list of projects is at **Annexure – III**.

Agenda 3: Medicinal Plant Research: The proposals of **18** research projects were placed before the committee. After detailed discussion, **06** proposals with Agenda items no. **3.2, 3.3, 3.5, 3.6, 3.7 & 3.8** were approved without any modification; **10** projects with Agenda items no. **3.1, 3.4, 3.9, 3.10, 3.11, 3.12, 3.13, 3.14, 3.15, 3.16, 3.17 & 3.18** were approved by the committee with the certain modifications and suggestions. The detailed list of projects is at **Annexure – IV**.

Agenda 4: Drug Standardization Research: The proposals of **13** Research projects were placed before the committee. After detailed discussion, all **13** projects from **Agenda Item No. 4.1 to 4.13** were approved by the committee without any modification. The detailed list of projects is at **Annexure – V.**

Agenda 5: Pharmaceutical Research: The proposals of **5** Research projects were placed before the committee. After detailed discussion, all **5** projects from **Agenda Item No. 5.1 to 5.5** were approved by the committee without any modification. The detailed list of projects is at **Annexure – VI.**

Agenda 6: Pharmacology Research: The proposals of **4** research projects were placed before the committee. After detailed discussion, 3 proposals with **Agenda item No. 6.2, 6.3 and 6.4** were approved without any modification. **1** project with **Agenda item No. 6.1** has been approved by the committee with certain modifications and suggestions. The detailed list of projects is at **Annexure – VII.**

Agenda 5: Clinical Research: The summaries of **21** Research projects under the head were placed before the committee. After detailed discussion, **6** proposals with Agenda items no. **7.1, 7.3, 7.5, 7.7, 7.8 & 7.20** were approved without any modification; 14 projects with Agenda items no. **7.2, 7.4, 7.9, 7.10, 7.11, 7.12, 7.13, 7.14, 7.15, 7.16, 7.17, 7.18, 7.19 and 7.21** were approved by the committee with the certain modifications and suggestions; and 01 projects with **Agenda item no. 7.6** was not discussed. The detailed list of projects is at **Annexure – VIII.**

Supplementary Agenda 1 (Literary Research): The proposal of 1 research project was placed before the committee. After detailed discussion, the project has been approved by the Committee.

At the end of meeting, Chairman PEMC - DG, CCRAS expressed sincere thanks to the members for extending their support, encouragement and offering valuable suggestions.

The meeting ended with vote of Thanks

ANNEXURE-I

List of Participants attended the Meeting on 2nd Project Evaluation Monitoring Committee (PEMC).

A. Members who attended the meeting

1.	**Prof. (Dr.) Rabinarayan Acharya, Director General,** CCRAS, New Delhi	**Chairman**
2.	**Dr. Atul Juneja,** Former Scientist E, email - atul_juneja@hotmail.com	**Member**
3.	**Prof. (Dr.) Suresh Kumar,** Dept. of Botany, Ramjas College,delhi email - suresh.kumar@ramjas.du.ac.in, drskumar35@gmail.com	**Member**
4.	**Dr. Rajeev Sharma,** Former Director, PCIM, Gaziabad,U.P, email - rajeevsharma.herb@gmail.com	**Member**
5.	**Dr. Sharad Srivastav,** Senior Principal Scientist and Head,Pharmacognosy Division, NBRI, Lucknow email - Sharad-ks@yahoo.co.in (online)	**Member**
6.	**Prof. Yogender Pal Khasa,** Department of Microbiology, University of Delhi, Delhi email - yogi110@gmail.com, ypkhasa@south.du.ac.in (online)	**Member**
7.	**Dr Sonia Khatter,** Professor(Microbiology), ESIC, Delhi, email - maliksonia@yahoo.com	**Member**
8.	**Dr. Gian Singh,** Former Scientist 'F' and Head, NISCAIR, Delhi, email - giansinghaulakh@gmail.com	**Member**
9.	**Dr. Virendra Singh Rana,** Principal Scientist, IARI, email - ranavs2000@yahoo.com	**Member**
10.	**Dr. S. K. Maulik,** Former professor, AIIMS, Delhi, email - subirmaulik@yahoo.com (online)	**Member**
11.	**Dr. Sharad Wakode,** Prof. (Pharmaceutical Chemistry), DIPSAR,Delhi, email -sharadwakode@gmail.com	**Member**
12.	**Dr. Galib, Associate Professor, AIIA, email** - Galib14@yahoo.co.in (online)	**Member**
13.	**Prof. Anil Singh,** Department of Dravyaguna, IMS,BHU, Varanasi, email-sn.anil@yahoo.com (online)	**Member**
14.	**Dr.Sudipt Rath,** Associate Professor ,Dept. of Dravyguna, email - sudipt@nia.edu.in,srath.nia@gov.in (online)	**Member**
15.	**Dr. M.M.Padhi,** Ex-DDG, CCRAS, Bhubaneswar, email - padhimm@gmail.com,padhimm@yahoo.co.in (online)	**Member**
16.	**Dr. Asit K. Panja,** Associate Professor, NIA, Jaipur, email - asitkpanja@gmail.com (online)	**Member**
17.	**Dr. Arun Gupta,** Professor Panchakarma, CBPACS, Delhi, email - arun24@hotmail.com (online)	**Member**
18.	**Prof. A.C.Kar,** Vikriti Vigyan, IMS, BHU, Varanasi, email - karanukul@rediffmail.com (online)	**Member**
19.	**Dr. N. Srikanth, DDG, CCRAS**	**Member Secretary**

B. Members who could not attend the meeting:

20.	**Mr. Raj Kumar,** Director (IFD), Ministry of Ayush, New Delhi.	**Member**
21.	**Dr. Sunita Garg, Former Principal Scientist, email - Sunita.niscair@gmail.com**	**Member**
22.	**Prof. B.J.Patgiri, (Dean Research), ITRA, Gujarat ,email - deanresearch@itra.edu.in**	**Member**

C. CCRAS Officers who attended the meeting

1. Dr. Sanjayakumar Y.R., Assistant Director (Pharmacology)
2. Dr. Ravinder Singh, Assistant Director (Chem.)
3. Dr. Adarsh Kumar, Assistant Director (Ay.)
4. Dr. B.C.S. Rao, Assistant Director (Ay.)
5. Dr. Pratap Makhija, Assistant Director (Ay.)
6. Dr. B. Venkateshwarlu, Assistant Director (Ay.)
7. Dr. Arjun Singh, Assistant Director (Chem.)
8. Dr. Deepa Makhija, Assistant Director (Ay.)
9. Dr. Renu Makhija, Assistant Director (Pathology)
10. Dr. Sarada Ota, Assistant Director (Ay.)
11. Dr. Sunita, Research Officer (Ay.)
12. Dr. B.S. Sharma, Research Officer (Ay.)
13. Dr. Vinod Kumar Lavaniya, Research Officer (Ay.)
14. Dr. Renu Singh Research Officer (Ay.)
15. Dr. A.K. Jain, Research Officer (Ay.)
16. Dr. Babita Yadav, Research Officer (Ay.)
17. Dr. Shiddamallayya, Research Officer (Botany)
18. Dr. Rakesh Kumar, Research Officer (Ay.)
19. Dr. Ashwathy Kutty, Research Officer (Ay.)
20. Dr. Lalita Sharma, Research Officer (Ay.)
21. Dr. Kalpana Kachare, Research Officer (Ay.)
22. Dr. Azeem Ahmad, Research Officer (Ay.)
23. Dr. Sophia, Research Officer (Ay.)
24. Dr. Bidhan Mahajon, Research Officer (Ay.)
25. Dr. Swati Sharma, Research Officer (Ay.)
26. Dr. Amit Rai, Research Officer (Ay.)
27. Dr. Pallavi Mundada, Research Officer (Ay.)
28. Dr. Anagha Ranade, Research Officer (Ay.)
29. Dr. R.K. Rana, Stat. Officer
30. Dr. Arunabh Tripathi, SO
31. Dr. Richa Singhal, SSA

D. CCRAS Officer who could not attend:

1. Dr. Shruti Khanduri Research Officer (Ay.)

ANNEXURE – II

Summary of completed and ongoing IMR/Collaborative Research Projects and outreach Activities during last five year & current year (2022-23)

S. No.	Research Projects	Projects Completed	Ongoing projects
1.	**Clinical Research including COVID-19 related Research initiatives**	94 Projects	44 Projects
2.	**Medicinal Plant Research**	20 Projects	29 Projects
3.	**Drug Standardization**	26 Projects	17 Projects
4.	**Pharmacological Research**	36 Projects	46 Projects
5.	**Literary Research**	25 Projects	18 Projects
6.	**Fundamental Research**	1 Project	4 Projects
7.	**Pharmaceutical Research**	1 Project	6 Projects
8.	**Systematic Review Projects**	30 Projects	-
9.	**Status of Research Oriented Public Health Care Programme (Outreach Activities)**		
	• Tribal Health Care Research Programme under TSP	• 61125 ST population covered<br>• 260206 ST Patients treated<br>• 1001 LHTs/ folk claims collected	
	• Ayurveda Mobile Health Care Programme under Scheduled Castes Sub Plan (SCSP)	• 26009 tour conducted<br>• 1158762 SC population surveyed<br>• 625627 SC Patients treated<br>• 7 Projects completed	
	• Reproductive & Child Health Care Programme under Scheduled Castes Sub Plan (SCSP)	• 8814 tour conducted<br>• 283250 SC population surveyed<br>• 160157 SC Patients treated<br>• 2 projects completed	
	• Integration of AYUSH (Ayurveda) component with NPCDCS (National Programme for Prevention and Control of Cancer, Diabetes, Cardiovascular diseases & Stroke) programme in the identified districts of 3 states viz. Bhilwara (Rajasthan), Surendranagar (Gujarat) and Gaya (Bihar)	• 111182 Patients Enrolled for selected non communicable diseases<br>• Overall encouraging benefits of standalone Ayurveda therapies as well as benefits as add-on therapies.<br>• Functional integration through delivering Ayurveda – Allopathy integrative heath care services in the management of non-communicable diseases	
	• Ayurvedic Health Centres under North East (NE) Plan of CCRAS	A total 581906 patients were attended.	

2. LITERARY RESEARCH

Minutes of the Projects discussed in 2nd PEMC held on 13th & 14th September, 2022

S. No.	Agenda No.	Title of the project	Name of Focus Group	Principal Investigator	Name of Participating Institute	Name of Programme Officer/ Nodal Officer/ Facilitator	Comments/ suggestions of PEMC	Approved or Not approved/ Deferred
1.	2.1	Translation of Turangaratnamala from Marathi to English	Focus group for Literary research	Dr.G.P.Prasad	NIIMH, Hyderabad	Dr. Adarsh Kumar/Dr.V.KLavaniya/Dr. Rakesh Narayanan V/Dr. Ashwathy kutty V	The project may be taken up as proposed	Approved
2.	2.2	Editing and Translation of Kitab fi al-Mizaj (A manuscript on Temperament) and Kitab fi al-Anasir (A manuscript on Constituents) written by Jālīnūs (d. 216 AD) from Arabic into English	Focus group for Literary research	Dr. Ashfaque Ahmed	NIIMH, Hyderabad	Dr. Adarsh Kumar/Dr.V.KLavaniya/Dr. Rakesh Narayanan V/Dr. Ashwathy kutty V	Projects mentioned at 2.2 and 2.3 may be merged in to one as they are of similar nature with provision for one SRF (Unani) and a tenure of two years.	Approved with modifications
3.	2.3	Editing and Translation of Qarabadin Masumi (A manuscript on pharmacopoeia) written by Masum al-Sheerazi (d. 1691 AD) from Persian into English	Focus group for Literary research	Dr. Ashfaque Ahmed	NIIMH, Hyderabad	Dr. Adarsh Kumar/Dr.V.KLavaniya/Dr. Rakesh Narayanan V/Dr. Ashwathy kutty V	Projects mentioned at 2.2 and 2.3 may be merged in to one as they are of similar nature with provision for one SRF (Unani) and a tenure of two years.	Approved with modifications
4.	2.4	Transcription, Editing and English Translation of 'Tibb-e-Shifa Mahmud Shahi' a Persian version of "Ashtāngahridaya",	Focus group for Literary research	Dr. Ashfaque Ahmed	NIIMH, Hyderabad	Dr. Adarsh Kumar/Dr.V.KLavaniya/Dr. Rakesh Narayanan V/Dr. Ashwathy kutty V	Publication of the manuscript may be taken up in first phase. The related references from various Ayurveda texts may be added as source references along with the Persian text. Budget may be revised as per norms.	Approved with modifications

ANNEXURE - IV

3. MEDICINAL PLANT RESEARCH

Minutes of the Projects discussed in 2nd PEMC held on 13th & 14th September, 2022

S. No.	Agenda No.	Title of the project	Name of Focus Group	Principal Investigator	Name of Participating Institute	Name of Programme Officer/ Nodal Officer/ Facilitator	Comments/ suggestions of PEMC	Approved or Not approved/ Deferred
1.	3.1.	Development and Digitalization of Botanical Reference Standards and Herbarium for the Medicinal Plants Appearing in Ayurvedic Formularies of India in Mandatory Drug Testing Laboratories of CCRAS	MPR-Pharmacognosy	Dr.G. Nartunai, Research Officer (Pharmacognosy)	CSMDRIA, Chennai	Dr.B.Venkateswarlu A.D. (Ay.)/Dr. Sidhamallayya R.O.(Bot.)	1. Change in the Project title BRS (Botanical Reference Standards) to Authentic Raw Drugs Samples (ARDS).<br>2. Parameters must be restricted to macro and microscopical studies.<br>3. Co-PI of the project should be reduced accordingly.<br>4. Revise the project budget accordingly.	Approved
2.	3.2.	Digitalization and indexing of herbarium sheets at CARI Kolkata collected from 1970-2000.	MPR-Medico Ethno Botanical Survey	Ms. Sreya Dutta R.O. (Botany)	CARI, Kolkata	Dr.B.Venkateswarlu A.D. (Ay.)/Dr. Sidhamallayya R.O.(Bot.)	The committee after detailed appraisal approved the proposal.	Approved
3.	3.3.	Medico Ethno Botanical Survey in the forest areas of Wayanad district of Kerala state	MPR-Medico Ethno Botanical Survey	Dr. Parvathy G Nair R.O. (Ayurveda)	NARIP, Cheruthuruthy	Dr.B.Venkateswarlu A.D. (Ay.)/Dr. Sidhamallayya R.O.(Bot.)	The committee after detailed appraisal approved the proposal.	Approved
4.	3.4.	"Medico Ethno Botanical Survey in the forest areas of Darjeeling district of West Bengal and Preparation of Monographs."	MPR-Medico Ethno Botanical Survey	Dr.Shriprakash, AD In-Charge (Ayurveda)	RARI, Gangtok	Dr.B.Venkateswarlu A.D. (Ay.)/Dr. Sidhamallayya R.O.(Bot.)	1. Title of the project to be changed as "Medico Ethno Botanical Survey in the forest areas of Darjeeling district of West Bengal".	Approved

5.	3.5.	Anthology of Survey Research Outcome from Medico Ethno Botanical Survey (MEBS), Central Ayurveda Research Institute, Jhansi.	MPR-Medico Ethno Botanical Survey	Dr. J.C. Arya, Research Officer (Bot.)	CARI, Jhansi	Dr.B.Venkateswarlu A.D. (Ay.)/Dr. Sidhamallayya R.O.(Bot.)	The committee after detailed appraisal approved the proposal.	Approved
6.	3.6.	Establishment of In vitro propagation protocol for the conservation of Hydnocarpus pentandrus (Buch.-Ham.) Oken, a Vulnerable medicinal plant used in Ayurvedic formulations	MPR-Cultivation	Dr. Arun M. Gurav, AD (Botany)	RARI, Pune	Dr.B.Venkateswarlu A.D. (Ay.)/Dr. Sidhamallayya R.O.(Bot.)	The committee after detailed appraisal approved the proposal.	Approved
7.	3.7.	Developing Quality Standards for selected Extra-pharmacopoeial Drugs Collected from Irula tribes of Thandarai, Kancheepuram District, Tamilnadu	MPR-Medico Ethno Botanical Survey	Dr. G. Kusuma, Research Officer (Ay.)	CSMDRIA, Chennai	Dr.B.Venkateswarlu A.D. (Ay.)/Dr. Sidhamallayya R.O.(Bot.)	The committee after detailed appraisal approved the proposal.	Approved
8.	3.8.	Developing image-based taxonomical database of plants used in the Ayurveda	MPR-Medico Ethno Botanical Survey	Dr. Rasika Kolhe, RO (Ayurveda)	RARI, Pune	Dr.B.Venkateswarlu A.D. (Ay.)/Dr. Sidhamallayya R.O.(Bot.)	The committee after detailed appraisal approved the proposal.	Approved
9.	3.9.	Development of quality standard of Medicinal plants used by name Pashan Bhed in different part of India.	MPR-Pharmacognosy	Dr. ShyamBaboo Prasad Research Officer (Pharmacognosy)	RARI, Pune	Dr.B.Venkateswarlu A.D. (Ay.)/Dr. Sidhamallayya R.O.(Bot.)	1. It is suggested to change the title of the project as "comparative study of Medicinal plants used by name PashanBhed in different parts of India." 2. Aflotoxins, Heavy metals and pesticide residues should be removed from the methodology of the project.	Approved with modifications
10.	3.10.	ComparativePharmacognostical and Phytochemical Standardization of Commonly Adulterating Ayurvedic Drugs and Their Adulterants.	MPR-Pharmacognosy	Dr. Rajesh Bolleddu RO (Pharmacognosy)	CARI, Kolkata	Dr.B.Venkateswarlu A.D. (Ay.)/Dr. Sidhamallayya R.O.(Bot.)	1. Conduct a Market survey and review the literature with the selected plants to study the	Approved with modifications

							comparative analysis of market samples.	
11.	3.11.	Development of Quality Standards of Selected Extra-pharmacopoeial Drugs (AnuktaDravyas) Used in Local Health Traditions Collected from Different Regions of India.	MPR-Pharmacognosy	Dr.G.Nartunai RO (Pharmacognosy)	CSMDRIA, Chennai	Dr.B.Venkateswarlu A.D. (Ay.)/Dr. Sidhamallayya R.O.(Bot.)	1. It is suggested to include markers study as per the availability of markers.	Approved
12.	3.12.	Development of Quality Standards of Selected Extra-pharmacopoeial Drugs (AnuktaDravyas) Used in Local Health Traditions Collected from Different Regions of India.	MPR-Pharmacognosy	Dr. Anupam K Mangal AD (Pharmacognosy)	CARI, Kolkata	Dr.B.Venkateswarlu A.D. (Ay.)/Dr. Sidhamallayya R.O.(Bot.)	1. It is suggested to include markers study as per the availability of markers.	Approved
13.	3.13.	Development of Quality Standards of Selected Extra Pharmacopoeial (Anukta Dravya) Drugs used in Local Health Traditions collected from different regions of India.	MPR-Pharmacognosy	Mrs. SapnaAvinashKondalkar RO (Pharmacognosy)	RARI, Gwalior	Dr.B.Venkateswarlu A.D. (Ay.)/Dr. Sidhamallayya R.O.(Bot.)	1. It is suggested to include markers study as per the availability of markers.	Approved
14.	3.14.	Development Of Quality Standards of Selected Extra-Pharmacopoeial Drugs (Anukt Dravyas) Used in Local Health Traditions Collected from Different Regions of India.	MPR-Pharmacognosy	Dr. ShyamBaboo Prasad RO (Pharmacognosy)	RARI, Pune	Dr.B.Venkateswarlu A.D. (Ay.)/Dr. Sidhamallayya R.O.(Bot.)	1. It is suggested to include markers study as per the availability of markers.	Approved
15.	3.15.	Antimicrobial Activity of selective Ayurveda formulations against Gastrointestinal disorder, Respiratory disorder and Urinary Tract Infection causing microorganisms.	MPR-Microbiology	Dr. Swamy CT, RO-Microbiology	CSMDRIA, Chennai	Dr.B.Venkateswarlu A.D. (Ay.)/Dr. Sidhamallayya R.O.(Bot.)	1. Title of the project to be changed as "Antibacterial Activity of selective Ayurveda formulations against Gastrointestinal infection, Respiratory infection and Urinary Tract Infection causing bacteria".<br>2. Revise the review of the literature with the bacterial growth references.	Approved

16.	3.16.	Evaluation of antimicrobial, anti-oxidant and immuno-modulatory properties of selected Ayurvedic formulations	MPR-Microbiology	Dr. Susmita Roy Research Officer (Microbiology)	CARI, Kolkata	Dr.B.Venkateswarlu A.D. (Ay.)/Dr. Sidhamallayya R.O.(Bot.)	1. Title of the project to be changed as "Evaluation of antibacterial, anti-oxidant and immuno-modulatory properties of selected Ayurvedic formulations". 2. Used methods like CLSC etc. are to be clearly defined in the methodology. 3. Define 2 broader/primary objectives & secondary objectives	Approved
17.	3.17.	To investigate the inhibitory effect of various Ayurvedic plants against dental caries causing micro-organisms.	MPR-Microbiology	Dr. Smriti Tandon (R.O. Microbiology)	CARI, Jhansi	Dr.B.Venkateswarlu A.D. (Ay.)/Dr. Sidhamallayya R.O.(Bot.)	1. Defined the methodology and procedure (Culture media references etc.) 2. Review the literature on each selected plant.	Approved
18.	3.18.	In vitro screening of plants/vegetables/fruits/herbs for their suitability as prebiotics.	MPR-Microbiology	Dr. Hemant Soni (R.O. Microbiology)	CARI, Jhansi	Dr.B.Venkateswarlu A.D. (Ay.)/Dr. Sidhamallayya R.O.(Bot.)	1. Define the criteria for the selection of plants. 2. Describe the Materials and methodology of the project. 3. The quality control is used for anaerobic bacteria and also biologica indicators.	Approved

ANNEXURE - V

4. DRUG STANDARDIZATION RESEARCH

Minutes of the Projects discussed in 2nd PEMC held on 13th & 14th September, 2022

S. No.	Agenda No.	Title of the project	Name of Focus Group	Principal Investigator	Name of Participating Institute	Name of Programme Officer/ Nodal Officer/ Facilitator	Comments/ suggestions of PEMC	Approved or Not approved / Deferred
1.	4.1.	QC Analysis, Estimation of Markers/PRS & Shelf-life Studies of Ayush PTK and Go-Mutra Haritaki	Focus group for Drug Standardization Research	Dr. Vijay Kumar & Dr. Arjun Singh	CARI, Jhansi	Dr. Ravindra Singh/Dr. Arjun Singh	Recommended as proposed	Approved
2.	4.2.	Effect of geographical variations, phytochemical profiling and marker-based quantification of selected medicinally important *Ficus* species.	Focus group for Drug Standardization Research	Dr. Vijay Kumar	CARI, Jhansi	Dr. Ravindra Singh/Dr. Arjun Singh	Recommended as proposed	Approved
3.	4.3.	Preclinical Standardization and Development of SOP for Coated Tablets from Aqueous and Hydroalcoholic Extracts of *Amlaki*, *Guduchi* and *Vasa* and Shelf-life Studies".	Focus group for Drug Standardization Research	Dr. Vijay Kumar Dr. Midhuna Mohan K.	CARI, Jhansi & RARI, Vijaywada	Dr. Ravindra Singh/Dr. Arjun Singh	Recommended as proposed	Approved
4.	4.4.	Marker-based Traceability in Ayush Kwath Churna and its Raw Drugs, Shelf-life and Exploration of Antioxidant & Antimicrobial Potency: A new horizon to explore Ayush Kwath Churna	Focus group for Drug Standardization Research	Mr. Hemant Rawat	CARI, Jhansi	Dr. Ravindra Singh/Dr. Arjun Singh	Recommended as proposed	Approved
5.	4.5.	Development of Quality Standards, Estimation of Markers and Shelf-Life Study of Ayush-82 and Ayush-SG"	Focus group for Drug Standardization Research	Mr. Hemant Rawat & Dr. Arjun Singh	CARI, Jhansi	Dr. Ravindra Singh/Dr. Arjun Singh	Recommended as proposed	Approved
6.	4.6.	Development of Drug Master File and Dossier for *Withania somnifera* (L.) Dunal an Important Medicinal Plant from Ayurveda and Contemporary Research".	Focus group for Drug Standardization Research	Dr. A.K.Meena	RARI, Gwalior	Dr. Ravindra Singh/Dr. Arjun Singh	Recommended as proposed	Approved
7.	4.7.	Comparative phytochemical and pharmacological evaluation of small branches with root or stem bark of selective Ayurvedic plants	Focus group for Drug Standardization Research	Dr. A.K.Meena	RARI, Gwalior	Dr. Ravindra Singh/Dr. Arjun Singh	Recommended as proposed	Approved

8.	4.8.	Rationalization of hydro-alcoholic extractive protocols for selected plant roots/rhizomes of Ayurvedic importance for strategic drug development".	Focus group for Drug Standardization Research	Dr. Deepak Kumar	CARI, kolkata	Dr. Ravindra Singh/Dr. Arjun Singh	Recommended as proposed	Approved
9.	4.9.	Development of Quality standards for formulation "AYUSH KVM Syrup	Focus group for Drug Standardization Research	Mrs. Manosi Das	CARI, kolkata	Dr. Ravindra Singh/Dr. Arjun Singh	Recommended as proposed	Approved
10	4.10.	Isolation of Phytoconstituents from some selected Ayurvedic medicinal plants.	Focus group for Drug Standardization Research	Mr. Kalyan Hazra	CARI, kolkata	Dr. Ravindra Singh/Dr. Arjun Singh	Recommended as proposed	Approved
11	4.11.	Estimation of Calcium and trace minerals & metals in Ayurveda drugs of Sudhavarga	Focus group for Drug Standardization Research	Dr. CH. Venkata Narasimhaji	CSMCARI, Chennai	Dr. Ravindra Singh/Dr. Arjun Singh	Recommended as proposed	Approved
12	4.12.	Development of quality control protocols, identification, and estimation of major bioactive constituents of Ayurvedic medicated oils 'Murivenna', 'DurvadiKera', 'BhringamalakadiKerataila'	Focus group for Drug Standardization Research	Dr. Parvathy G Nair	NARIP, Cheruthuruthy	Dr. Ravindra Singh/Dr. Arjun Singh	Recommended as proposed	Approved
13	4.13.	QC analysis and estimation of markers in polyherbal formulation Jwarahara kwatha Choornam (JKC) for the effective treatment of fever	Focus group for Drug Standardization Research	Mr. Thirupataiah B.	NARIP, Cheruthuruthy	Dr. Ravindra Singh/Dr. Arjun Singh	Recommended as proposed	Approved

5. PHARMACEUTICAL RESEARCH

Minutes of the Projects discussed in 2nd PEMC held on 13th & 14th September, 2022

S. No.	Agenda No.	Title of the project	Name of Focus Group	Principal Investigator	Name of Participating Institute	Name of Programme Officer/ Nodal Officer/ Facilitator	Comments/ suggestions of PEMC	Approved or Not approved / Deferred
1.	5.1	Process validation, Pharmaceutical standardization and evaluation of antimicrobial activity of Ajeernakantak Ras, Kanaksundar Ras and Hinguleshvara Ras.	Drug Standardization	Dr. Chandrashekhar Jagtap,	CARI-Jhansi	Dr. Ravindra Singh<br>Dr. B.S. Sharma<br>Dr. Shruti Khanduri	The project proposal may be taken up as proposed	Approved
2.	5.2	Process validation, Pharmaceutical standardization with classical and modern techniques alngwith the evaluation of shelf life and antimicrobial activity of Pippalyadi Yonivarti, Arkadi Yonivarti and Kushthadi Yonivarti.	Drug Standardization	Dr. Neelam Kumari Singh	CARI-Jhansi	Dr. Ravindra Singh<br>Dr. B.S. Sharma<br>Dr. Shruti Khanduri	The project proposal may be taken up as proposed	Approved
3.	5.3	Process validation and Pharmaceutical Standardization of Polyherbal formulations (Ajmodadi Churna, Panchkola Churna and Haridradi Churna) with its shelf life evaluation.	Drug Standardization	Dr. Neelam Kumari Singh	CARI-Jhansi	Dr. Ravindra Singh<br>Dr. B.S. Sharma<br>Dr. Shruti Khanduri	The project proposal may be taken up as proposed	Approved
4.	5.4	Pharmaceutical standardization, antimicrobial activity and Accelerated stability study of the arka of Ajomoda Guduchi, Kiratatikta and Mishreya.	Drug Standardization	Dr. Deepa Sharma	CARI-Jhansi	Dr. Ravindra Singh<br>Dr. B.S. Sharma<br>Dr. Shruti Khanduri	The project proposal may be taken up as proposed	Approved
5.	5.5	Development of SOP and quality standards of Ayurvedic herbo-mineral formulation Gandhaka Rasayana.	Drug Standardization	Dr. Supna Saha	CARI, Kolkata	Dr. Ravindra Singh<br>Dr. B.S. Sharma<br>Dr. Shruti Khanduri	The project proposal may be taken up as proposed	Approved

6. PHARMACOLOGY RESEARCH

Minutes of the Projects discussed in 2nd PEMC held on 13th & 14th September, 2022

S. No.	Agenda No.	Title of the project	Name of Focus Group	Principal Investigator	Name of Participating Institute	Name of Programme Officer/ Nodal Officer/ Facilitator	Comments/ suggestions of PEMC	Approved or Not approved/ Deferred
1.	6.1	Anti-cancer activity of Chaulmoogra oil and Shwetha Sarshapa churna in the treatment of epidermal carcinoma of skin in experimental mice	Pharmacological research (Pre Clinical Studies)	Dr. S. Chitra, Assistant Director (Biochemistry)	CSMCARI- Chennai	Dr. Sanjaya Kumar Y.R, AD (Pharmacology)	1.It was suggested to carry out In vitro studies in the cell lines at first instance. Based on the leads from the in vitro studies, invivo studies may be taken up.<br>2. One Co-Investigator from RARI-Pune may be included in the project so as to facilitate In vitro cell line studies.	Approved with modification
2.	6.2	Safety and anti dyslipidemic activity of Tryusanadi Guggulu in experimental animals	Pharmacological research (Pre Clinical Studies)	R. Ilavarasan Assistant Director (Pharmacology)	CSMCARI- Chennai	Dr. Sanjaya Kumar Y.R, AD (Pharmacology)	1.It was suggested to modify the title of the project as 'Safety and anti obesity activity of Ayurvedic formulation Tryusanadi Guggulu in experimental animals'.	Approved

3.	6.3	Study of coded drugs intended for antenatal care i.e. AYUSH-GG and AYUSH-AG for their developmental toxicity and teratogenicity profiles in zebrafish embryo model	Pharmacological research (Pre Clinical Studies)	Dr. Brijesh S. Sisodia Assistant Director (Biochemistry)	RARI-Gwalior	Dr. Sanjaya Kumar Y.R, AD (Pharmacology)	1. Principal Investigator of the project was advised to undergo training to obtain advanced expertise in zebra Fish models.	Approved
4.	6.4	Anticancer Potential and Molecular Mechanism of Rasa sindhoor on Ovarian Cancer Cells	Pharmacological research (Pre Clinical Studies)	Dr. Lalrin Puia, M.VSc.<br><br>Research Officer (Pharmacology) & Dr. Amit Kumar Srivastava, Ph.D.<br><br>Scientist & Ramalinga Fellow	CARI-Kolkata & CSIR-IICB, Kolkata	Dr. Sanjaya Kumar Y.R, AD (Pharmacology)	1. The project proposal was approved without any modification.	Approved

7. CLINICAL RESEARCH

Minutes of the Projects discussed in 2nd PEMC held on 13th & 14th September, 2022

S. No.	Agenda No.	Title of the project	Name of Focus Group	Principal Investigator	Name of Participating Institute	Name of Programme Officer/ Nodal Officer/ Facilitator	Comments/ suggestions of PEMC	Approved or Not approved/ Deferred
1.	7.1.	Efficacy of Ayurveda Interventions (Pushkar Guggulu and Haritaki) as add-on to Standard Care in Stable Coronary Artery Disease: A Double-blind Randomized Controlled Study	Cardiovascular	1.Dr. Anunay Gupta Department of Cardiology, VMMC and Safdarjung Hospital, New Delhi 2.Dr. Sakshi Sharma, RO (Ay.), CARI Delhi	CARI Delhi	Program Officer: Dr. BCS Rao Nodal officer: Dr Babita Yadav	---	Approved
2.	7.2.	A randomized controlled clinical trial to evaluate the efficacy of ayurveda treatment protocol in lumbar disc herniation with radiculopathy	Panchkarma	Dr. Aswani. P.S, R.O.(Ay.) NARIP, Cheruthuruthy	NARIP, Cheruthuruthy	Program Officer: Dr. BCS Rao Nodal officer: Dr Babita Yadav	Inclusion criteria: The reference score for inclusion as per the Oswestry Disability Index may be added	Approved with minimal suggestions
3.	7.3.	Efficacy and safety of Murivenna anal infiltration compared to Diltiazem topical application in chronic anal fissure: A prospective randomized open-label clinical trial	Ano-rectal Diseases	Dr. K M Pratap Shankar, R.O.(Ay.)	NARIP, Cheruthuruthy	PO: Dr. BCS Rao Nodal officer: Dr Babita Yadav	---	Approved
4.	7.4.	Efficacy of Ayurveda interventions (Hridyarnava Rasa and Harityakyadi yoga) as an add-on to standard care in Stable Coronary Artery Disease (CAD) assessed through Global Longitudinal Strain Imaging Technique (GLSIT) - A Randomized Controlled Trial	NA	1. Dr. Rohit Madhav Sane, Madhavbaug 2. Dr. Laxman Bhurke	CARI Mumbai	Program Officer: Dr. BCS Rao Nodal officer: Dr Babita Yadav	Urine heavy metals and USG abdomen may also be included for safety assessment.	Approved with minor amendment

5.	7.5.	A cross sectional study to assess the prevalence of Metabolic Syndrome and its association with Prakriti and NCD risk factors in the adult population of South Andaman district, Andaman & Nicobar Islands.	Life style disorders	Dr. Akash Lal	RARI, Port Blair	Dr. Azeem Ahmad	-	Approved
6.	7.6.	A prospective randomized clinical trial to assess the efficacy and safety of Arjunarista and Navaka guggulu in Dyslipidemia as a preventive measure for vascular disorders						Not discussed
7.	7.7.	A Randomized Controlled Clinical Trial to Compare the Efficacy of Individualized Ayurveda Therapeutic Regimen and Physiotherapy on Functional Disability and QoL in Post Stroke Rehabilitation	NA	Dr. Remya E.	NARIP, Cheruthuruhty	Dr. Babita yadav<br>Dr. Sophia	-	Approved
8.	7.8.	An exploratory series of n of 1 responder restricted study of Ayurveda regimen on quality of life among elderly population in Ballabhgarh district of Haryana- A community based study	Nil	Dr Punit Misra, AIIMS	AIIMS, Ballabhgarh	Dr Shruti Khanduri	-	Approved
9.	7.9.	A Clinical Consensus study for developing the practice guidelines of Marsa Nasya Karma with a multi-center Feasibility study in Cervical Spondylosis	Focus group for Pancakarma	Dr.Devi R Nair	NARIP, Cheruthuruthy	Dr. B.C.S Rao	Suggested<br>1.To elaborate the inclusion criteria adding the details given under the section of diagnostic criteria<br>2. To remove the second point of the inclusion criteria ie the participants who are willing to participate in the study.	Approved with certain modifications
10.	7.10.	Topical Oil Pooling (Karnapurana) with Kshirabala Taila and suppleMentation of Ashwagandha Churna	Shalakya	Dr. Krishna Kumar V, R.O. (Ay.)	NARIP, Cheruthuruthy	Dr. Pallavi Mundada	1) Justification of the trial interventions may be provided<br>2) The title of	Study was approved with suggested revisions

		(TOPMAC) in presbycusis – collaborative pilot randomized controlled trial					the study may be revised as "Efficacy of Topical Oil Pooling (Karnapurana) with Kshirabala Taila and suppleMentation of Ashwagandha Churna (TOPMAC) in presbycusis – An exploratory randomized controlled trial"<br>3) The study duration is not appropriate as compared to the proposed sample size. The recruitment period may be reduced to 12 months and the budget may be revised accordingly	
11.	7.11.	Exploring the Association between the type of Prakriti (Body Constitution) and Prevalence of Diabetic Retinopathy (DR): A Cross- Sectional Study "	Shalakya	Dr. Bhavya, R.O. (Ay.)	CARI, Bengaluru	Dr. Pallavi Mundada	1) The study title may be revised as "A cross-sectional survey to explore the association of prakriti with diabetic retinopathy"<br>2) Detailed justification for sample size calculation may be provided<br>3) The study endpoint is inappropriate and may be revised<br>4) The primary outcomes may be deleted and re-written as "type of prakriti of the diabetic retinopathy cases"<br>5) Secondary	Study was approved with suggested revisions

								outcome may be re-written as "Prevalence of other risk factors based on Ayurveda philosophy, associated with Diabetic retinopathy"<br>6) Based on the sample size proposed, one contractual research staff may be proposed and the budget and the recruitment period may be revised accordingly<br>7) Incidence of adequate number of DR cases may be ensured to complete the survey in one year post preparatory period.	
12.	7.12.	A Randomized controlled trial to evaluate the efficacy of Uttarabhaktikasnehapana in the management of Migraine	Shalakya	Dr. Parvathy G Nair	NARIP, Cheruthuruthy	Dr. Pallavi Mundada	1) The budget may be presented in the format of CCRAS research policy<br>2) The proposed sample size is unclear. Whether the total sample size is 40 or 80 may be specified with appropriate justification in consultation with the Biostats section of CCRAS.<br>3) If the sample size is 80, a contractual research staff may be proposed to complete the recruitment in at least 15	Study was approved with suggested revisions

							months and the budget may be revised accordingly<br>4) The objectives may be redrafted scientifically.<br>5) In the secondary outcome measures, either VAS or NRS (any one) may be proposed<br>6) The outcome measures may be redrafted scientifically<br>7) The study title may be revised to "A Randomized controlled trial to evaluate the efficacy of Uttarabhaktika snehapana in reducing frequency of Migraine headaches"	
13.	7.13.	Clinical evaluation of Ayurvedic management in Allergic Rhinitis-A Collaborative Randomized Controlled trial	Shalakya	Dr. Shweta Mata, R.O. (Ay.)	CARI, New Delhi	Dr. Pallavi Mundada	1) The study duration is not appropriate as compared to the proposed sample size. The recruitment period may be reduced to 15 months and the budget may be revised accordingly<br>2) The duration of Follow-up may be increased to at least 6 months for the said condition<br>3) The objectives and the outcome measures may be redrafted scientifically<br>4) The inclusion and exclusion	Study was approved with suggested revisions

							criteria are not clearly mentioned. They may be revised	
14.	7.14.	Evaluation of the efficacy and safety of Chandraprabha vati with Gokshuradi Guggulu in the management of Benign Prostatic Hyperplasia- A randomized, standard controlled clinical trial	Urinary disorders and gout related research	Dr. Shah Aaditya Bhavanbhai	RARI, Jammu	Dr. Renu Singh<br>Dr. Lalita Sharma	➢ Proper justification for choice of drugs may be included in the proposal<br>➢ Change in serum PSA level may be removed from secondary outcome as the subjects with normal PSA are to be included in the study.	Approved with minor suggestions
15.	7.15.	Clinical evaluation of integrating Yoga module with Ayurvedic regimen (Mild purgation and Phalaghrita) and conventional medicine (Letrozole) in the management of unexplained and anovulatory female infertility; a Randomized open label comparative trial		Dr. Anubha Chandla	RARI, Mandi	Dr. Sarada Ota	1. The title may be revised as "Efficacy of Ayurveda regimen (mild purgation and internal oleation) along with yoga module in the management of unexplained and anovulatory female infertility – A randomized controlled trial"<br>2. The primary objective of the study may be revised as "To assess the efficacy of Ayurveda	Study was approved with suggested modifications

							regimen along with Yoga module in the management of female infertility<br>3. Justification of the selected intervention may be provided<br>4. Sample size may be recalculated with proper justification<br>5. Detail protocol of the Yoga module (with duration of each Yoga intervention) may be provided.<br>6. Mantra chanting may be specified (e.g. om chanting) with duration<br>7. Yoga module may be practiced throughout the week (at least 5 days/week)<br>8. The office assistant/DEO post may be replaced by Yoga instructor	
16.	7.16.	Clinical evaluation of Ayurvedic formulations in the management of Raktapradara w.s.r. menorrhagia – A collaborative randomized controlled clinical trial		Dr. Shivshankar Rajput, R.O. (Ay.)	CARI, New Delhi (CRU, Safdarjung Hospital)	Dr. Sarada Ota	1. The study title may be revised as "Efficacy of Ayurveda interventions in the management of Menorrhagia (*Raktapradara*) – A randomized	Study was approved with suggested modifications

							controlled trial"<br>2. Justification for the selection of the study interventions may be provided<br>3. The objectives may be revised as per the revised title. Clinical safety may be removed from the secondary objectives<br>4. Sample size may be recalculated with proper justification<br>5. The inclusion and exclusion criteria should be revised and stated more clearly. [E.g. Duration of the condition (menorrhagia) may be specified in the inclusion criteria.]<br>6. The diagnostic criteria are not appropriate. This may be revised based on the condition selected<br>7. The dose of IFA may be verified and revised as per availability in the	

							market. (Folic acid may not be available in the currently mentioned dose.)	
17.	7.17.	To assess the efficacy of PK Avalehya and Bala taila Abhyanga in Sutika Paricharya (w.s.r to management of puerperium)- A Randomized open label controlled clinical study		Dr. U R Sekhar Namburi	Not mentioned	Dr. Sarada Ota	1. The study title may be revised as "To assess the role of *PK avaleha* and *Bala taila abhyanga* in peurperium management (*Sutika paricharya*) – A randomized controlled trial<br>2. The study centre (from where the participants will be recruited) may be specified<br>3. Justification for the role of the investigators mentioned in the proposal may be given<br>4. The sample size may be recalculated with proper justification<br>5. The budget table may be revised. Ayush AG row to be deleted. Panchakarma attendant post may be replaced with MTA post<br>6. Office assistant/DEO may be removed.	Study needs major modifications.

							7. 02 SRFs may be demanded based on the sample size<br>8. The study methods and SOPs may be described in detail (e.g. Bala taila abhyanga will be started from which day of delivery)<br>9. The participants will be recruited after how many days after delivery is not mentioned in the Inclusion criteria<br>10. Justification for the non-recurring items and the conventional medicines may be provided in detail<br>11. The proposed budget seems exaggerated so proper justification for each head of the demanded budget may be provided<br>12. Serum prolactin may be removed from the list of outcome measures<br>13. The study objectives and outcomes measures and how they will be assessed	

							should be rewritten scientifically and revised based on the study title 14. Control group interventions are not mentioned anywhere. Describe the interventions with proper justification	
18.	7.18.	Evaluation of the efficacy of Ayush-BR Leham in the management of Moderate malnutrition in children aged 3-5 years: a randomized controlled open label prospective clinical study		Dr. Karthika Dr. Renu Rani More investigators may be added	CARI, New Delhi	Dr. Sarada Ota	1) Identification of the study centres (Anganwadi Kendra) 2) A multi-centre study may be proposed after exploring the feasibility of execution and the budget may be revised accordingly 3) The title of the study may be revised as "*Evaluation of the efficacy of Ayush BR Leham as adjuvant to the standard of care in the management of moderate malnutrition among children aged 3 to 5 years from Anganwadi Centres – A multi-centre randomized controlled preliminary trial*"	Study was approved with suggested modifications

19.	7.19.	Impact of Mukta Shukti Bhasma and Saubhagya Shunti in a reversal of Low Bone density mass in Lactating women consuming traditional foods: A randomized prospective clinical trial		Dr. Lalita Savardekar, NIRRH, Mumbai & Dr. Kuldeep Choudhary, CARI Mumbai	ICMR-NIRRH, Mumbai & CARI, Mumbai	Dr. Sarada Ota	1. The study title may be revised as "Impact of *Mukta Shukti Bhasma* and *Saubhagya Shunti* in reversal of low bone mass density among Lactating women consuming traditional foods: A randomized controlled preliminary trial"<br>2. The mode of collaboration and budget allocation at both study centres may be defined in the proposal.<br>3. Provide the justification for the proposed sample size.<br>4. The trial drugs and the investigations head in the budget may be revised to make it more realistic.	Study was approved with suggested modifications
20.	7.20.	A Randomized Double blind Placebo control clinical study to evaluate the immunomodulatory effect of Swarnprashan in moderately malnourished children	RCH with main focus on Paediatrics	Dr. Vikas Agarwal, Deptt. Of Immunology, SGPGIMS, Lucknow	CCRAS in collaboration with Sanjay Gandhi post graduate institute of medical sciences, Lucknow	Dr. B.C.S. Rao, A.D. (Ay.)/Dr. BabitaYadav/ Dr. Swati Sharma	Project was thoroughly discussed and approved	Approved

21.	7.21.	Clinical study to evaluate the efficacy and safety of an Ayurvedic interventions in sickle cell disease-An add on Randomized Controlled Trial	NIL	Dr. M.N. Suryawanshi	1.Datta Meghe Institute of Medical Sciences, Wardha<br>2. RARI, Nagpur.	• Program Officer-Dr.Deepa Makhija<br>• Nodal Officer-Dr Kalpana Kachare	The sample size may be recalculate from statistical section	Project Approved with a recommendation to change in duration of treatment of the intervention group same as the control group. i.e. change intervention for 8 months in place of 6 months. There is no requirement for follow-up without treatment.

ANNEXURE - IX

Supplementary Agenda – 1: LITERARY RESEARCH

Minutes of the Projects discussed in 2nd PEMC held on 13th & 14th September, 2022

S. No.	Agenda No.	Title of the project	Name of Focus Group	Principal Investigator	Name of Participating Institute	Name of Programme Officer/ Nodal Officer/ Facilitator	Comments/ suggestions of PEMC	Approved or Not approved/ Deferred
1.	S.A. 1	Translation of unpublished manuscript Shalihotra from Sanskrit to English	Focus group for Literary research	Dr.G.P.Prasad	NIIMH, Hyderabad	Dr. Adarsh Kumar/Dr .V.KLavaniya/Dr. Rakesh Narayanan V/Dr. Ashwathy kutty V	The project was recommended for approval in principle, However, the budget may revised and put before the DG, CCRAS in the capacity of chairman of PEMC	Technically approved
